# Peoria Posterior Fossa Stroke Scale: A New Scale to Assess the Severity of Posterior Fossa Stroke Symptoms and Outcomes

**DOI:** 10.7759/cureus.94115

**Published:** 2025-10-08

**Authors:** Lisle W Blackbourn, Deepak Nair, Arun Talkad

**Affiliations:** 1 Neurology, University of Illinois College of Medicine Peoria, Peoria, USA; 2 Neurology, OSF Illinois Neurological Institute, Peoria, USA

**Keywords:** national institutes of health stroke scale, nihss, peoria posterior fossa stroke scale, posterior circulation stroke, posterior fossa stroke, stroke, stroke scale

## Abstract

Introduction: Posterior fossa strokes often present with symptoms that are less obvious and can overlap with other conditions. While the National Institutes of Health Stroke Scale (NIHSS) remains a valuable tool for stroke assessment, its limitations in posterior fossa strokes necessitate a complementary approach. A posterior fossa stroke scale developed with standard protocols for use in various settings, such as pre-hospital, emergency department, and inpatient care, could ensure early identification and timely treatment. For this reason, the Peoria Posterior Fossa Stroke Scale (PPFSS) was created with the objective of creating an add-on to the NIHSS to allow for the assessment of the possibility of a posterior fossa stroke by any healthcare provider.

Materials and methods: A literature review of peer-reviewed publications via PubMed was conducted to identify clinical signs and symptoms associated with posterior fossa strokes and existing posterior fossa stroke scales. A preliminary scale draft was created and formatted for easy bedside application, which was then discussed with a panel of five vascular fellowship-trained neurologists and later a neuro-ophthalmologist to create a finalized scale.

Proposed scale: The PPFSS consists of a six-section scoring system with a range of scores of zero to 15 and includes sections that include eyes, nystagmus, head impulse, hearing, pharynx, and balance that would be used in the acute setting of those suspected to have posterior fossa strokes.

Limitations and future directions: Further research into the PPFSS would need to be done to validate its use. This would include testing in real-world settings to determine the sensitivity and specificity of PPFSS in identifying posterior circulation strokes, inter-rater reliability as measured by Cohen’s kappa, and comparison to other scoring systems. Cognitive dysfunction of the patient and interrater reliability may factor into the scoring of the PPFSS.

Conclusion: A standardized, easy-to-use approach for assessing posterior fossa strokes could ensure consistency in diagnosis and treatment across healthcare settings. The PPFSS was developed as a potentially usable scale at bedside in the acute stroke setting that is ready for a validation research study.

## Introduction

The National Institutes of Health Stroke Scale (NIHSS) is a widely used tool for assessing the severity of stroke symptoms and predicting outcomes [1]. While it is highly effective for anterior circulation strokes, its application and value in posterior fossa strokes present challenges. Posterior fossa strokes often present with symptoms that are less obvious and can overlap with other conditions. Symptoms such as vertigo, ataxia, and visual disturbances may not be well assessed by the NIHSS, which was designed to assess anterior circulation strokes [2-5]. This is highlighted in the fact that higher median scores have been shown to occur in anterior circulation strokes compared to posterior when using the NIHSS [3]. The NIHSS does not thoroughly evaluate cerebellar signs or brainstem symptoms, which are crucial for diagnosing posterior strokes [2-5]. For example, patients who have isolated diplopia or vertigo without significant motor deficits can lead to an underestimation of stroke severity in the patient. Further, patients with posterior fossa strokes might have difficulty communicating their symptoms, such as dizziness or visual disturbances, which are not directly assessed by NIHSS [2-5]. This limitation can affect the accuracy of the scale in representing the true extent of impairment and, therefore, lead to an underestimation of the stroke’s impact on patient function and prognosis. Studies have shown that a suspected 20-60% of acute ischemic strokes are missed in the emergency room setting [2]. It has also been shown that posterior fossa strokes are almost three times as likely to be missed when compared to anterior circulation strokes.

While the NIHSS remains a valuable tool for stroke assessment, its limitations in posterior fossa strokes necessitate a complementary approach. For more comprehensive evaluations, clinicians might integrate other scales or other diagnostic methods alongside the NIHSS to better address the unique challenges posed by posterior circulation strokes. However, current scales, such as the Posterior NIHSS (POST-NIHSS), which have been made to assess posterior fossa strokes, have their own limitations [6]. These scales are not equipped to be scored in an acute stroke setting, with parts of the score having been assessed by a speech therapist within 48 hours of symptom onset and not by the physician. Further, the POST-NIHSS dysphagia component was not assessed using the same screening tool in all patients. A more standardized assessment could ensure consistency in diagnosis and treatment across healthcare settings. There have been other scales, such as the Adam’s Scale of Posterior Stroke (ASPOS), that attempt to solve this issue. ASPOS, however, has its own problems in discriminating eye symptoms [7]. Validation research showed that the "Eyes" category was not statistically significant when determining discrimination value. This alone can be an important section for the ability to assess and evaluate posterior fossa strokes. 

The implementation of a posterior fossa stroke scale that could be used in an acute setting has the potential to significantly improve stroke outcomes through early and accurate diagnosis, targeted treatment, and better resource utilization, ultimately enhancing patient care and reducing the burden of stroke-related complications. For this reason, the Peoria Posterior Fossa Stroke Scale (PPFSS) was created with the objective of creating an add-on to the NIHSS to allow for the assessment of the possibility of a posterior fossa stroke. Further, the objective was to create a scoring system that could be used by any healthcare provider while focusing on improving issues in current scoring systems.

## Materials and methods

The PPFSS was created by assessing the limitations of the NIHSS in posterior fossa strokes, as well as previously created stroke scales that assess posterior fossa strokes. The goal was to create a scoring system for use in various settings, such as pre-hospital, emergency department, and inpatient care, that could ensure early identification and timely treatment of posterior fossa strokes that addressed these limitations. Since the NIHSS should be done on every stroke patient, it was further determined to create a scale to be implemented as an add-on to the NIHSS to allow for the assessment of the possibility of a posterior fossa stroke by any healthcare provider without duplication of information.

A literature review of peer-reviewed publications via PubMed was conducted to identify clinical signs and symptoms associated with posterior fossa strokes and any existing posterior fossa stroke scales. Identifying clinical signs was done by searching "clinical symptoms posterior fossa stroke" and "posterior fossa stroke signs" and limiting results to the last 10 years when completed in June of 2024. No Boolean terms were used. This resulted in 134 and 224 articles, respectively. Articles were not reviewed if related to the pediatric population or surgery-focused. Only free full-text articles in English were reviewed. This was to identify possible symptoms to include in the scale to discuss with a panel of vascular fellowship-trained neurologists. To identify possible current scales to use as a comparison, PubMed was searched using the term "posterior stroke scale" with a limit to the last five years, yielding 765 studies. Not all scales found were used. Some results of this search were also used for clinical signs to consider.

Each item then chosen was defined with clear descriptors and a binary or graded scoring system to reduce subjectivity. Each category grading system subscale was determined by the possible severity of each symptom in a posterior fossa stroke. A preliminary scale draft was created and formatted for easy bedside application. At this time, the scoring system was discussed with a panel of five vascular fellowship-trained neurologists to determine possible gaps in the scale and potential issues non-neurologic providers might have using the PPFSS. Each member of the panel was blinded to the others' comments and opinions on the scale. Comments and opinions were then compared to existing literature, and scales were found to try to determine from known data what would give the best sensitivity and specificity. The scale modifications after this were then discussed with a neuro-ophthalmologist for a different perspective. A final scale was then made after this.

The final PPFSS is a structured, rapid-assessment tool designed in theory for use by any healthcare provider. It complements the NIHSS by focusing on features of posterior fossa stroke not assessed in the NIHSS and aims to minimize diagnostic delays in this challenging patient population.

## Results

Proposed scale

The PPFSS consists of a six-section scoring system with a range of scores of zero to 15 (Table 1).

**Table 1:** Scoring sections and ranges for the Peoria Posterior Fossa Stroke Score. EOM: extraocular movements; INO: internuclear ophthalmoplegia

Peoria Posterior Fossa Stroke Scale		
Category	Score	Exam Findings
Eyes	0	Normal pupils and EOM
	1	Pupillary asymmetry w/o abnormal EOM
	2	Unilateral ophthalmoparesis/plegia
	3	INO or "one-and-a-half-syndrome"
Nystagmus	0	No nystagmus
	1	Any nystagmus with eye movement
	2	Direction-changing nystagmus OR gaze-evoked vertical nystagmus
	3	Vertical nystagmus spontaneously in primary gaze
Head Impulse	0	No nystagmus OR abnormal head impulse
	1	Normal head impulse
Hearing	0	Normal
	1	New unilateral hearing loss
Pharynx	0	No tongue/uvular deviation and normal cough AND/OR gag reflex
	1	Tongue OR uvular deviation with preserved cough AND/OR gag reflex
	2	Weak cough AND/OR gag reflex regardless of tongue/uvular deviation
	3	Absent cough AND/OR gag reflex
If safe to attempt ambulation, then…		
Balance	0	Normal stance and gait
	1	Imbalance while walking
	2	Swaying with standing AND/OR broad-based stance
	3	Imbalance/Falling while standing
	4	Truncal ataxia while sitting
Peoria Posterior Fossa Stroke Scale Score: 0 to 15		

The NIHSS should be done on all suspected stroke patients. The PPFSS was therefore made as an add-on to the NIHSS, and therefore, anything examined in the NIHSS was excluded from the PPFSS, such as motor strength testing. Both scoring systems should be used in the situation of a suspected or confirmed posterior fossa stroke. It is the belief that higher scores are associated with a higher probability of a stroke located in the posterior fossa. All sections are made to be able to be assessed bedside in an acute scenario and assess for potential stroke symptoms arising from the posterior fossa. The fact that the physical exam might not be completed by a neurologist, or even a physician, was also considered when creating the scoring system.

Sections include eyes, nystagmus, head impulse, hearing, pharynx, and balance. The head impulse section should only be done if nystagmus is present in the patient. The balance section should only be done if it is safe to ambulate the patient. Further, once the patient scores points in the balance section, testing balance should be stopped. Further details of each section are described below.

Eyes

This section refers to the eye range of motion and pupil size of the patient. Scores range from zero to three, with zero equaling normal pupils and extraocular movements (EOM). A score of one would have pupillary asymmetry without abnormal EOM, a score of two would have unilateral ophthalmoplegia, and a score of three would have internuclear ophthalmoplegia (INO) or “one-and-a-half” syndrome. Infarcts in the brainstem can lead to a range of clinical eye issues, depending on the location of the infarct in the brainstem, with the most clinically obvious eye movement issue being INO, although other disorders can cause this clinical syndrome [8,9].

Nystagmus

Nystagmus should be assessed when testing extraocular movements in all directions and in primary gaze. A score of zero would be in a patient with no nystagmus. A score of one would be any nystagmus seen that does not fall into a score of two or three, and was added for ease of scoring for those in specialties that are not neurology. For example, simple fatigable nystagmus was a consideration of potential confounding scores, so this category was made to help differentiate the type of nystagmus seen and the potential for a posterior fossa stroke. A score of two would be for direction-changing nystagmus or gaze-evoked vertical nystagmus, and a score of three would be for vertical nystagmus spontaneously seen in primary gaze. These nystagmus patterns have been shown to be highly sensitive to stroke [10,11].

Head Impulse

Head impulse would be done only if the patient was scored for nystagmus. If no nystagmus is present, the patient would score a zero in this section, as the head impulse would not need to be completed. If nystagmus is present, head impulse testing should be done. A score of zero would also be marked if the head impulse was abnormal, indicating a peripheral cause of the nystagmus [10,12]. A score of one would be given if the head impulse was normal, indicating a central cause of the nystagmus [10,12]. This section would require further teaching of medical treatment team members, such as bedside nurses and paramedics, to perform correctly. Situations might arise where it is unsafe to perform the head impulse, such as occipital craniotomy or suspected cervical injury. In cases like this, it should be assumed to have the same score from the previous exam and not be performed again. This section was created from the HINTS exam to help distinguish between central and peripheral causes of acute vestibular syndrome [10]. The Head-Impulse-Nystagmus-Test-of-Skew (HINTS) exam, however, is only useful in that scenario and cannot be applied to broader posterior fossa stroke symptoms.

Hearing

For the hearing section, scoring must exclude chronic hearing loss and bilateral hearing loss. A score of zero would be given for the above situations and normal hearing. A score of one would be given in situations of new unilateral hearing loss. This does not have to be a completely absent hearing.

Pharynx

The pharynx section would evaluate the cough reflex, the gag reflex, tongue deviation, and uvular deviation. A score of zero would have all the above intact with no abnormalities. A score of one would have tongue or uvular deviation with preserved cough and gag reflex. A score of two would be given if the patient had a weak cough and/or gag reflex, regardless of tongue or uvular deviation. Lastly, a score of three would be given for absent cough and/or gag reflex. Only one of the cough or gag reflexes being tested would be sufficient for scoring.

Balance

The last section of the scale would only be done if it is safe to ambulate the patient. Examination should start with testing truncal ataxia and lead up to walking to safely assess the patient. Once the patient scores points, testing balance should be stopped. A score of zero should be given for normal stance and gait. A score of one would be given for imbalance while walking. A score of two would be given for swaying with standing with eyes open and/or a broad-based stance. A score of three would be scored for imbalance/falling while standing. Lastly, four would be given for truncal ataxia while sitting. Imbalance is one of the most common symptoms of cerebellar strokes, but it can also be seen in peripheral disorders and thus should have a way to assess the imbalance severity across a range of scores [13].

## Discussion

The determination of included categories was done based on what was thought to have objective measurements on physical exam. For this reason, other potential posterior fossa stroke symptoms like dizziness and nausea were excluded, as they were determined to be more subjective and might not be able to be seen on exam. It was also felt that dizziness might be captured in the "balance" section or the "ataxia" section of the NIHSS. Although a patient complaining of acute-onset dizziness might be a situation where the PPFSS should be used clinically.

Limitations and future directions

Stroke remains a clinical diagnosis, and the PPFSS should not be the determining factor of a diagnosis for a patient. Similar to the NIHSS, the PPFSS would be a means to assess the severity of stroke symptoms and predict outcomes. The main limitation of the PPFSS is no patient population data or testing, making the PPFSS not currently validated and hypothesis-generating. The PPFSS would need to be tested to validate the scale in the acute clinical stroke setting. Differences in scores between posterior fossa strokes versus other causes of symptoms, along with testing how scores improve over time with clinical improvement of the patients, would need to be assessed. Currently, this makes the PPFSS purely speculative. 

By implementing and collecting further data, numerical thresholds for the determination of sensitivity and specificity of identifying a posterior fossa stroke, along with potential quantification of stroke severity and/or identification of posterior fossa strokes, could occur.

Cognitive dysfunction of the patient or acute encephalopathy situations have the potential to create inaccurate scoring, no different than when the NIHSS is attempted in these patients. Inter-rater reliability might also be a factor, especially considering the exam might be performed by emergency medicine providers to neurologists. Recognizing INO or performing a head impulse has the greatest potential for issues in this regard. If shown to be a validated scale, education or standardized training for this population would need to be done for proper scoring in these sections. This could include in-person teaching or video lessons showing correct technique.

Future validation

The following outlines a proposed pilot study to validate the PPFSS. As this would require using the PPFSS on patients, IRB approval would be required for any such study, whether retrospective or prospective. The PPFSS will be applied alongside the NIHSS at first contact by a neurologist. All adult patients (>18 years) presenting to the ED or assessed in the hospital setting with acute neurologic symptoms less than 24 hours from symptom onset and with symptoms suggestive of stroke, particularly those involving dizziness, imbalance, visual changes, or ataxia, will be eligible. Patients with clear alternative diagnoses (e.g., intoxication, seizure) at the time of presentation will be excluded; however, if the diagnosis is still unclear and posterior fossa stroke is still in the differential, then they would still be included. Although stroke is a clinical diagnosis, imaging modalities such as MRI should be used to determine whether the patient has a posterior stroke versus a mimicking diagnosis. Given that the PPFSS would be done prior to such imaging, this would naturally blind the examiner when scoring to the end diagnostic outcome of the patient. If imaging was done prior to PPFSS being performed, then these patients should be excluded. Primary outcome measurements could include the sensitivity and specificity of PPFSS in identifying posterior circulation strokes. Secondary outcomes might include comparison of PPFSS accuracy to NIHSS in posterior fossa stroke cases, as well as inter-rater reliability as measured by Cohen’s kappa.

Other scales

The two main posterior fossa stroke scales are the POST-NIHSS and ASPOS. In comparison, the PPFSS attempts to solve key issues in each. The PPFSS was made in an attempt to help discriminate eye symptoms more easily, as well as be assessed in an acute stroke setting. The NIHSS should already be done in any stroke patient. The PPFSS would then be done to complement the NIHSS by focusing on features of posterior fossa stroke not assessed in the NIHSS, with the aim of minimizing diagnostic delays in this challenging patient population. However, validation of the PPFSS is needed.

## Conclusions

The NIHSS remains a valuable tool for stroke assessment; however, its limitations in posterior fossa strokes necessitate a complementary approach. Other scales and scoring systems have been made to assess posterior fossa strokes but have their own limitations, including not being fully completed in an acute stroke setting. For these reasons, the PPFSS was created as a structured scale with bedside usability by various healthcare providers to assess posterior fossa strokes. The PPFSS was created as an add-on to the NIHSS while trying to address shortcomings seen in other scoring systems. Further research with IRB approval into the PPFSS would need to be done to ensure potential differentiation between posterior fossa strokes versus other causes of symptoms and to be able to assess clinical improvement in patients. This would include validation research, sensitivity and specificity analysis, inter-rater reliability testing, and potentially multicenter trials. A standardized, easy-to-use approach for assessing posterior fossa strokes in the acute setting, such as potentially the PPFSS, could ensure consistency in diagnosis and treatment across healthcare settings while improving early recognition. In summary, the PPFSS is a proposed framework ready for future validation research for use in posterior fossa strokes.
